# Microglial TNFα Induces COX2 and PGI2 Synthase Expression in Spinal Endothelial Cells during Neuropathic Pain

**DOI:** 10.1523/ENEURO.0064-17.2017

**Published:** 2017-04-21

**Authors:** Hirosato Kanda, Kimiko Kobayashi, Hiroki Yamanaka, Masamichi Okubo, Koichi Noguchi

**Affiliations:** Department of Anatomy and Neuroscience, Hyogo College of Medicine, Nishinomiya, Hyogo 663-8501, Japan

**Keywords:** Endothelial cells, Microglia, Neuropathic pain, PGI2, Spinal cord, TNFα

## Abstract

Prostaglandins (PGs) are typical lipid mediators that play a role in homeostasis and disease. They are synthesized from arachidonic acid by cyclooxygenase 1 (COX1) and COX2. Although COX2 has been reported to be upregulated in the spinal cord after nerve injury, its expression and functional roles in neuropathic pain remain unclear. In this study, we investigated the expression of Cox2, PGI2 synthase (Pgis), and prostaglandin I2 receptor (IP receptor) mRNA in the rat spinal cord after spared nerve injury (SNI). Levels of Cox2 and Pgis mRNA increased in endothelial cells from 24 to 48 h after nerve injury. IP receptor mRNA was constitutively expressed in dorsal horn neurons. A COX2 inhibitor and IP receptor antagonists attenuated pain behavior in the early phase of neuropathic pain. Furthermore, we examined the relationship between COX2 and tumor necrosis factor-α (TNFα) in the spinal cord of a rat SNI model. Levels of TNFα mRNA transiently increased in the spinal microglia 24 h after SNI. The TNF receptors Tnfr1 and Tnfr2 mRNA were colocalized with COX2. Intrathecal injection of TNFα induced Cox2 and Pgis mRNA expression in endothelial cells. These results revealed that microglia-derived TNFα induced COX2 and PGIS expression in spinal endothelial cells and that endothelial PGI2 played a critical role in neuropathic pain via neuronal IP receptor. These findings further suggest that the glia–endothelial cell interaction of the neurovascular unit via transient TNFα is involved in the generation of neuropathic pain.

## Significance Statement

This study reports the expression of Cox2, PGIS, and IP receptor mRNA and proteins in the rat spinal cord following spared nerve injury. We found that Cox2 and Pgis increased in endothelial cells from 24 to 48 h after nerve injury, and a COX2 inhibitor and IP receptor antagonists attenuated pain behavior in the early phase of neuropathic pain. Moreover, our findings indicate that microglia-derived TNFα induced COX2 and PGIS expression in spinal endothelial cells and had a critical role in endothelial PGI2 via neuronal IP receptor in neuropathic pain. These findings suggest that the activation of glia–endothelial cell interaction by transient TNFα and endothelial cell–derived COX2-PGI2 has a modulatory effect in the generation of neuropathic pain.

## Introduction

Peripheral nerve injury may induce neuropathic pain with intense allodynia and hyperalgesia. Peripheral and central sensitization are pivotal pathomechanisms of neuropathic pain and lead to the development of a variety of pain behaviors (Basbaum et al., 2009; von Hehn et al., 2012). Recent findings on the mechanisms of neuropathic pain showed that activated glial cells in the spinal cord begin to secrete proinflammatory mediators, including tumor necrosis factor-α (TNFα), interleukin (IL)-1β, and IL-6, and may modulate excitability and sensitivity in the nociceptive pathway (Clark et al., 2013; Ji et al., 2013), resulting in neuropathic pain. How these factors secreted from glial cells affect nociceptive neurons in the spinal cord has not yet been fully elucidated.

Peripheral nerve injury causes significant changes in blood vessels in the spinal cord and leads to a decrease in tight junctions of the blood–spinal cord barrier (BSCB; Echeverry et al., 2011), modulation of BSCB permeability (Beggs et al., 2010), and infiltration of immune cells into the spinal cord (Zhang et al., 2007; Cao and DeLeo, 2008). In the brain, inflammatory cytokines have been reported to increase blood–brain barrier (BBB) permeability (Shen et al., 2009; Echeverry et al., 2011; Daneman, 2012; Alvarez et al., 2013), and TNFα-induced BBB disruption was inhibited by treatment with a cyclooxygenase (COX) inhibitor (Candelario-Jalil et al., 2007). COX1 and COX2 metabolize arachidonic acid to prostaglandin (PG) H2, which is a common substrate for different prostanoid synthases. COX1 is known as a constitutive enzyme, and COX2 is inducible by inflammation and encoded by an immediate-early gene (Dubois et al., 1998; Turini and DuBois, 2002). COX2 is one of the important enzymes in processes leading to fever and inflammatory pain conditions (Turini and DuBois, 2002; Ricciotti and FitzGerald, 2011).

One of the prostanoids, PGI2 (known as a prostacyclin), is synthesized by PGI2 synthase (PGIS) produced in endothelial cells and has a variety of effects on the cardiovascular system, such as potent vasodilation and stimulation of platelet aggregation (Majed and Khalil, 2012). PGI2 binds to the G protein–coupled IP receptor, which is coupled to Gs, Gq, or Gi protein (Lawler et al., 2001). It has been reported that IP receptor mRNA is expressed in the dorsal root ganglion (DRG) and that PGI2 plays a pivotal role in pathologic pain and inflammatory responses (Oida et al., 1995; Murata et al., 1997; Bley et al., 1998; Schuh et al., 2013).

We have previously reported that peripheral nerve injury induced the upregulation of Cox2 in blood vessels of the ipsilateral spinal cord (Kanda et al., 2013). At that time, the detailed function of COX2 in blood vessels, the mechanism underlying inducible COX2 expression, and the relevance of the pathogenesis of neuropathic pain were unknown. In the current study, we investigated the expression of COX2, PGI2, and IP receptor in the spinal cord after spared nerve injury (SNI) and examined an inducing factor of COX2 and PGIS. We suggest a three-sided relationship model for a mechanism that contributes to neuropathic pain, describing the interactions among microglia, endothelial cells, and neurons in the spinal cord, and the role of TNFα as a messenger molecule from activated microglia.

## Methods

### Animal treatment

All animal experiments conformed to the regulations of the Hyogo College of Medicine Committee on Animal Research and were performed in accordance with the guidelines of the National Institutes of Health on animal care. Every effort was made to minimize animal suffering and reduce the number of animals used. Male Sprague-Dawley rats weighing 200–250 g were anesthetized with sodium pentobarbital (50 mg/kg, i.*p*.), and the tibial and common peroneal nerves were transected, while the sural nerve was left intact (SNI model). The wounds were closed, and the rats were allowed to recover. At several time points after surgery (12, 18, 24, 48 h and 3, 7, 14, 30 d), groups of rats were processed for analysis.

### Reverse-transcription PCR and *in situ* hybridization histochemistry

The rats were killed by decapitation under deep ether anesthesia. They were transcardially perfused with PBS, and their spinal cords (L4–5) were dissected out on a cold plate (0–4°C). The spinal cord was divided into left (ipsilateral side) and right (contralateral side) parts by a sagittal cut, rapidly frozen with powdered dry ice, and stored at –80°C until used. The extraction of total RNA was conducted using the RNA extraction reagent Isogen (Nippon Gene), and the PCR reaction was performed as described before (Kobayashi et al., 2006). PCR primers for *Cox2*, *Pgis*, *IP receptor*, *TNFα*, *Tnfr1, Tnfr2 receptors*, and *GAPDH* cDNA were designed as follows. *Cox2* primers (accession number AF233596): sense 5′-GGGTGTCCCTTCGCCTCTTT-3′; antisense, 5′-GTTGCCGGTATCTGCCTTCA-3′; *Pgis* primers (accession number U53855): sense, 5′-GGTGACCGCCTTCTCCTCTT-3′; antisense, 5′-GGTACCGGATGGGCACATCT-3′; *IP receptor* primers (accession number XM_218457): sense, 5′-GGACTGAGGGACTTCAGAAG-3′; antisense, 5′-GCCATACCCTGCCACTCTCT-3′; *TNFα* primers (accession number X66539): sense, 5′-GCCCACGTCGTAGCAAACCA-3′; antisense, 5′-GGGCTCATACCAGGGCTTG-3′; *Tnfr1* primers (accession number M63122): sense, 5′-CCCCAGGGAAAGTATGCCCA-3′; antisense, 5′-CCACTGGGGATATCGGCACA-3′; *Tnfr2* primers (accession number AY191269): sense, 5′-CCCCCTGGCCAGTATGCAAA-3′; antisense, 5′-GGGCTCTGGCTGAGATACGT-3′; and *GAPDH* primers (accession number M17701): sense, 5′-CCAGGGCTGCCTTCTCTTGT-3′; antisense, 5′-CCAGCCTTCTCCATGGTGGT-3′. The PCR reaction was performed using a standard method described elsewhere (Kobayashi et al., 2006). For *in situ* hybridization histochemistry (ISHH), the rats were killed by decapitation under deep ether anesthesia. The bilateral L4–5 spinal cord was dissected out, rapidly frozen in powdered dry ice, and cut on a cryostat to a thickness of 12–16 µm. Sections were thaw-mounted onto MAS-coated glass slides (Matsunami) and processed for ISHH as described before (Kobayashi et al., 2006, 2008). Data were expressed throughout as mean ± SEM (%). Differences in changes of values over time of each group were tested using one-way ANOVA, followed by individual *post hoc* comparisons (Fisher’s). A difference was accepted as significant when *p* < 0.05.

### Immunohistochemistry

The rats were killed by decapitation under deep ether anesthesia. The bilateral L4–5 spinal cord was dissected out, rapidly frozen in powdered dry ice, and cut on a cryostat to a thickness of 12 µm. Sections were thaw-mounted onto MAS-coated glass slides and fixed in 0.4% or 4% formaldehyde in 0.1 m phosphate buffer (PB; pH 7.4) for 10 or 20 min, respectively. After washing in TBS (0.1 m Tris-HCl, pH 7.4, and 0.15 m NaCl), the sections were immersed in 50% ethanol for 10 min, 70% ethanol for 10 min, and 50% ethanol for 10 min to enhance antibody penetration. For single immunohistochemistry (IHC) staining of COX2, the sections were preincubated in TBS containing 10% normal horse serum (NHS) for 30 min, followed by incubation in goat anti-COX2 (M-19) polyclonal antibody (1;1000 and 1:2500, Santa Cruz, sc-1747, 0.1 mg/mL, RRID: AB_2084976) containing 5% NGS overnight at 4°C. This COX2 antibody has been widely used in previous studies (Konsman et al., 2000, 2004; Yamagata et al., 2001; Nadjar et al., 2005; Inoue et al., 2006). A preabsorption control with the COX2 (M-19) peptide (corresponding to the C terminus of COX2 of mouse origin, Santa Cruz, sc-1747-p, 0.2 mg/mL) was performed to test the specificity of the anti-Cox2 antibody. Anti-COX2 IgG was incubated in five times its weight of COX2 peptide (0.5 or 0.2 µg/mL) for 2 h at room temperature. After incubation, the preabsorbed antibody was reacted with the spinal cord section for incubation overnight at 4°C. The sections were washed in TBS and incubated in biotinylated anti-goat IgG (1:200; Vector Laboratories) in TBS containing 5% NHS overnight at 4°C, followed by incubation in avidin-biotin peroxidase complex (Elite ABC kit; Vector) for 1 h at room temperature. The horseradish peroxidase reaction was developed in TBS containing 0.05% DAB (Wako) and 0.01% hydrogen peroxidase. The sections were then washed in TBS and dehydrated in a graded ethanol series, cleared in xylene, and coverslipped. For double immunofluorescence of COX2 with NeuN or PECAM1, the slices were incubated with a mixture of primary antibodies. The following antibodies were used: goat anti-COX2 (M-19) and polyclonal antibody (1:200, 1:500, Santa Cruz), mouse anti-NeuN monoclonal antibody (1:2000, EMD Millipore Bioscience Research Reagents, MAB377 clone A60, RRID: AB_2298772), and mouse anti-PECAM1 (CD31) monoclonal antibody (1:250, EMD Millipore MAB1393 clone TLD-3A12, RRID: AB_2161017). A mixture of corresponding secondary antibodies with donkey anti-mouse IgG Alexa Fluoro 488 (1:1000; Invitrogen) and biotinylated anti-goat IgG were incubated overnight at 4°C. Sections were then incubated in streptavidin conjugated to Alexa Fluor 568 (1:5000; Invitrogen) for 1 h at room temperature. These slides were rinsed with TBS and coverslipped with ProLong Gold anti-fade reagent with DAPI (Invitrogen).

### Double immunohistochemistry

The rats were deeply anesthetized with sodium pentobarbital and perfused transcardially with 250 ml of 1% paraformaldehyde (PFA) in 0.1 m PB, pH 7.4, followed by 500 ml of 4% PFA in 0.1 m PB. The spinal cords were dissected out and postfixed in the same fixative at 4°C overnight, followed by immersion in 20% sucrose in 0.1 m PB at 4°C for 2 d. The bilateral L4–5 spinal cord was dissected out, rapidly frozen in powdered dry ice, and cut on a cryostat to a thickness of 30 µm. Free-floating sections were washed in TBS (0.1 m Tris-HCl, pH 7.4, and 0.15 m NaCl) and immersed in 50% ethanol for 10 min, 70% ethanol for 10 min, and 50% ethanol for 10 min to enhance antibody penetration. For double immunofluorescence of Iba1 with phospho ERK1/2 or phospho p-38, the slices were incubated in a mixture of primary antibodies. The sections were preincubated in TBS containing 10% normal donkey serum for 30 min, followed by incubation in rabbit anti-phospho-ERK1/2 (Thr202/Tyr204) polyclonal antibody (1:500, Cell Signaling Technology, 9101, RRID: AB_331646), rabbit anti-phospho p-38 (Thr180/Tyr182) polyclonal antibody (1:1000, Cell Signaling Technology, 9211, RRID:AB_331641), and goat anti-Iba1 polyclonal antibody (1:500). After washing in TBS, a mixture of corresponding second antibodies with donkey anti-rabbit IgG Alexa Fluoro 488 (1:1000; Invitrogen) and anti-goat IgG Alexa Fluoro 568 (1:1000; Invitrogen) were incubated at 4°C overnight. These slides were rinsed with TBS, coverslipped with ProLong Gold antifade reagent with DAPI (Invitrogen), and digitized with a Nikon Eclipse E800 microscope connected to a Nikon DS-Ri1 digital camera.

### Double-labeling with IHC and ISHH

To examine the distribution of mRNA levels of Cox2, Pgis, IP receptor, and Tnfr, we used a double-labeling method, combining IHC with ISHH. The fresh-frozen sections were fixed in 0.4% (for COX2 staining) or 4% (for NeuN, GFAP, Iba1, and PECAM1 staining) formaldehyde in 0.1 m PB for 20 min. The following antibodies were used: rabbit anti-ionized calcium binding adaptor molecule 1 (Iba1) polyclonal antibody (1:100, Wako Chemicals), mouse anti-NeuN monoclonal antibody (1:1000, EMD Millipore Bioscience Research Reagents), rabbit anti-glial fibrillary acidic protein (GFAP) polyclonal antibody (1:1000, Dako, RRID: AB_10013382), mouse anti-PECAM1 (CD31) monoclonal antibody (1:250, EMD Millipore), and goat anti-COX2 (M-19) polyclonal antibody (1:5000, Santa Cruz). The treatment of sections and the method of double-labeling with IHC and ISHH were described in a previous article (Kobayashi et al., 2006).

### Drug treatments

#### COX2 inhibitor and IP receptor antagonist treatment

One day before SNI surgery, the L5 vertebrae were laminectomized under adequate anesthesia with 4% isoflurane, and a 7-cm soft tube (Silastic laboratory tubing, Dow Corning Corporation; outer diameter, 0.64 mm) filled with saline was inserted into the subarachnoid space for a length of 0.5 cm. After the muscle incision was closed, the tube was fixed, and the cut end was ligated. The tube was laid under the skin, and the incision was closed. 1, 2, or 7 d after SNI, the rats were anesthetized, and drugs were injected using a Hamilton syringe. The COX2 inhibitor NS398 (5 µg/10 µl, 50 µg/10 µl), or the IP receptor antagonist CAY10441 (2 µg/10 µl, 20 µg/10 µl) was carefully injected in volumes of 10 µl followed by 5 µl saline. The concentration of NS398 was 5 µg/10 µl and 50 µg/10 µl diluted in 70% dimethyl sulfoxide (DMSO); the concentration of CAY10441 was 2 µg/10 µl and 20 µg/10 µl diluted in 20% DMSO. Various concentrations of DMSO in saline were used as vehicle control. The doses of these drugs were chosen based on previous studies (Yamamoto and Nozaki-Taguchi, 1996; Ma et al., 2002; Muramatsu et al., 2012; Takahashi et al., 2013).

#### MAP kinase inhibitor treatments

Soft tube implantation surgery was performed at the same time as SNI surgery. Mini-osmotic pumps were implanted (Alzet model 1003D; 3d pump, 1 µl/h) and filled with one of the following drugs; 0.5 µg/µl MAPK kinase (MEK) 1/2 inhibitor, U0126 (1,4-diamino-2,3-dicyano-1,4-bis(2-aminophenylthio) butadiene; Calbiochem) in 50% DMSO, and 0.5 µg/µl p38 MAPK inhibitor, SB203580 (4-(4-fluorophenyl)-2-(4-methylsulfinylphenyl)-5-(4-pyridyl) 1H-imidazole; Calbiochem) in 50% DMSO. The doses of these drugs were chosen based on previous studies (Kobayashi et al., 2012).

#### Recombinant TNFα injection

After a 2-d recovery period from tube surgery, the rats were anesthetized, and 50 ng/10 µl recombinant rat TNFα (R&D Systems) in PBS or PBS alone was injected using a Hamilton syringe. The dose was chosen based on previous studies (Cao et al., 1998). Three hours after administration, the rats were killed by decapitation under deep ether anesthesia, and semiquantitative RT-PCR and ISHH were performed.

#### Neutralizing antibody treatments

After tubing surgery, mini-osmotic pumps were implanted and filled with one of the following drugs: 0.25 µg/µl hamster anti-mouse/rat TNF-α IgG clone TN3-19.12 (BD PharMingen, 557516, RRID: AB_398625; Sheehan et al., 1989; Lambertsen et al., 2009) in PBS or 0.25 µg/µl purified NA/LE hamster IgG1 λ1 isotype control (BD PharMingen, 553951, RRID: AB_395155) in PBS.

### Photomicrographs

All emulsion-coated slides and DAB-stained slides were digitized with a Nikon Eclipse 80i microscope connected to a Nikon DXM-1200F digital camera. Optical images were acquired using a FV1200 BX61 confocal microscope (Olympus). Confocal *z*-stacks, consisting of nine optical sections 0.58 µm apart (total *z*-depth of 5.22 μm) were taken with a dry objective lens (UPLSAPO 40× 2, N.A. = 0.95, Olympus). *z*-projection of stack series was summed using ImageJ (RRID: SCR_003070). We used Adobe Photoshop CS4 or Element 12.0 (Adobe Systems) to optimize images and to create all figures.

### Behavioral tests

All SNI rats were tested for mechanical allodynia and hyperalgesia of the plantar surface of the hindpaw before indwelling tube surgery, 1 d before SNI and 1, 2, or 7 d after surgery. Mechanical allodynia was assessed with a dynamic plantar aesthesiometer (Ugo Basile). To measure mechanical thresholds of the hindpaw, rats were placed in a plastic cage with a wire mesh floor and allowed to acclimate for 15 min before each test session. A paw-flick response was elicited by applying an increasing force (measured in grams) directly on the lateral portion of the plantar surface of the ipsilateral hindpaw (sural nerve territory). The force applied was initially below the detection threshold, then increased from 1 to 50 g in 0.1-g steps over 10 s, and was then held at 50 g for an additional 10 s. The rate of force increase was 5 g/s. The force required to elicit a reflex removal of the ipsilateral hindpaw was monitored. The amount of force required was defined as the mean of three measurements made at 5-min intervals. Data are expressed as mean ± SD. Differences in changes of values over time of each group were tested using one-way ANOVA, followed by individual *post hoc* comparisons (Fisher’s PLSD test). A difference was accepted as significant if *p* < 0.05.

## Results

### Expression of COX2, PGIS, and IP receptor in the spinal cord and the effects of peripheral nerve injury

**Figure 1.**
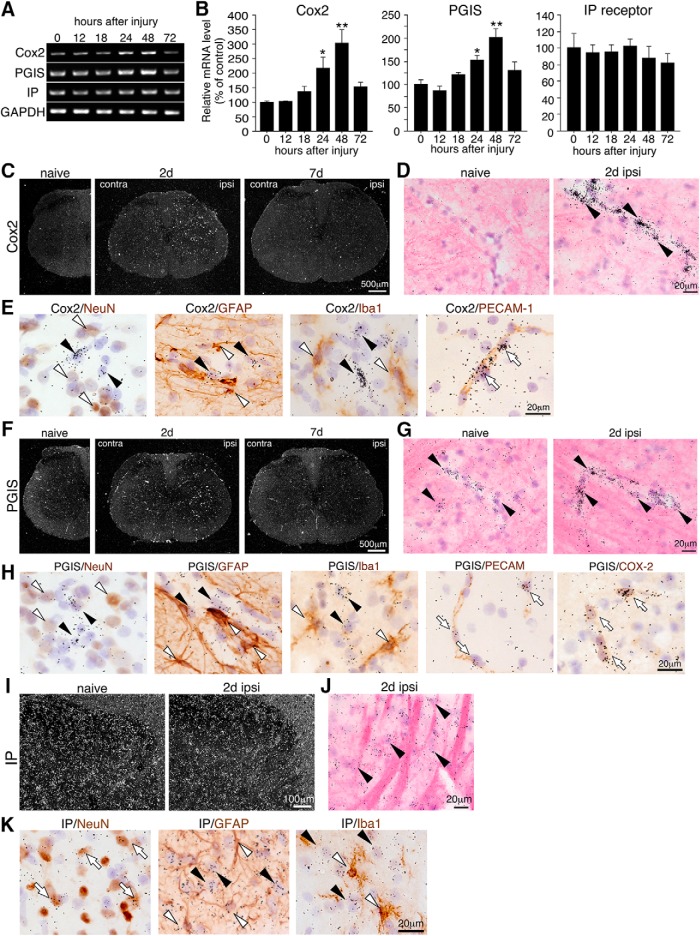
SNI up-regulated Cox2 and Pgis mRNA in the ipsilateral spinal cord. ***A***, Semiquantitative RT-PCR analysis showed Cox2, Pgis, and IP receptor mRNA expression in the L4-L5 ipsilateral spinal cord taken from rats at 0 (naive), 12, 18, 24, 48, and 72 h after nerve injury. GAPDH was used as the loading control. ***B***, Quantification of the relative mRNA levels of Cox2, Pgis, and IP receptor mRNA. The data were first normalized to GAPDH and then expressed as fold change compared with the naive rat spinal cord (*n* = 5, mean ± SE; *, *p* < 0.05; **, *p* < 0.001, Fisher’s PLSD). ***C–K***, ISHH images of the spinal cord. Dark-field images of ISHH revealed mRNA distribution of Cox2 (***C***), Pgis (***F***), and IP receptor (***I***) after peripheral nerve injury in the L4–5 spinal cord. Scale bar: (***C***, ***F***) 500 µm, (***I***) 100 µm. Higher-magnification photographs from hematoxylin and eosin–counterstained sections under bright-field illumination in the ipsilateral dorsal horn for Cox2 (***D***), Pgis (***G***), and IP receptor (***J***) of naive rats or 2 d after nerve injury. Arrowheads indicate positive cells. Scale bar: 20 µm. ***E***, Bright-field photomicrographs showed combined ISHH for Cox2 mRNA with immunostaining of NeuN, GFAP, Iba1, and PECAM1. ***H***, Pgis mRNA with immunostaining of NeuN, GFAP, Iba1, PECAM1, and COX2. ***K***, IP receptor mRNA with immunostaining of NeuN, GFAP, and Iba1, counterstained with hematoxylin, in the dorsal horn 2 d after nerve injury. Open arrowheads indicate single immunostained cells (brown staining). Black arrowheads indicate cells single-labeled with ISHH (aggregation of grains). Open arrows indicate cells double-labeled with ISHH and IHC. Scale bar: 20 µm. 2 d: 2 days (48 h) after surgery. Ipsi, ipsilateral side; contra, contralateral side.

We first used a semiquantitative reverse-transcription (RT)-PCR method to examine the effects of peripheral nerve injury on the expression of Cox2, Pgis, and IP receptor mRNA in the spinal cord (Fig. 1*A*). Comparisons were made against the naive rat L4–5 spinal cord. Cox2 and Pgis mRNA levels in L4–5 of the ipsilateral spinal cord showed a significant increase from 24 h and reached a peak 48 h after nerve injury (Cox2, 302.3% ± 46.5%; Pgis, 202.2% ± 10.2%, *p* < 0.001 versus naive; Fig. 1*B*). By 72 h after injury, Cox2 and Pgis mRNA levels had returned to basal values. There was no significant change in IP receptor mRNA levels in the spinal cord after peripheral nerve injury (Fig. 1*B*).

We then examined the distribution of Cox2, Pgis, and IP receptor mRNA (Fig. 1*C–K*) using ISHH. The intensity of the specific signal for Cox2 mRNA was very low in naive rats. Forty-eight hours after injury, Cox2 mRNA signals significantly increased in the ipsilateral spinal cord but completely decreased to naive levels by day 7 (Fig. 1*C*). Bright-field images showed that the signals for Cox2 mRNA after SNI were reported mainly in blood vessels (Fig. 1*D*), which is consistent with our previous report (Kanda et al., 2013). To characterize Cox2 mRNA–expressing cells in the spinal cord after nerve injury, we performed combined ISHH for Cox2 mRNA with IHC for NeuN, a marker for neurons; GFAP, a marker for astrocytes; Iba1, a marker for microglia; or PECAM1 (CD31), a marker for endothelial cells at 48 h after injury (Fig. 1*E*). Induced Cox2 mRNA was primarily colocalized with endothelial cells, but rarely with neurons, astrocytes, or microglia.

We detected weak Pgis mRNA signals in the spinal cord of naive rats (Fig. 1*F*). Nerve injury leads to an increase in the density of silver grains, indicating Pgis mRNA in the ipsilateral spinal cord after 48 h, but signals had returned to normal intensity by day 7 (Fig. 1*F*). Bright-field images showed that the Pgis mRNA signal was detected in blood vessel–like cells in naive rats and at 48 h after injury (Fig. 1*G*). To characterize Pgis mRNA–expressing cells in the spinal cord after nerve injury, we performed ISHH-IHC double-labeling. Induced Pgis mRNA was colocalized with PECAM1 but not with NeuN, GFAP, or Iba1 (Fig. 1*H*), suggesting that PGIS is expressed in endothelial cells, but not neurons, astrocytes, or microglia 48 h after nerve injury. Furthermore, induced COX2 was completely colocalized with Pgis mRNA in endothelial cells (Fig. 1*H*). These results indicate that PGI2 is synthesized in endothelial cells via COX2 after nerve injury.

We then investigated the expression of the PGI2 receptor, IP, after nerve injury. IP receptor mRNA was broadly expressed in the spinal dorsal horn (Fig. 1*I*), and bright-field images showed the aggregation of silver grains in cells with large nuclei that were lightly stained by hematoxylin in the dorsal horn 2 d after SNI (Fig. 1*J*). To identify the cell types expressing IP receptor mRNA, we performed ISHH-IHC double-labeling. We found signals of IP receptor mRNA in cells labeled with NeuN but not with GFAP or Iba1 (Fig. 1*K*). These results indicate that the PGI2 receptor is located in spinal neurons, and that PGI2 from endothelial cells could affect spinal neurons after peripheral nerve injury.

### COX2 immunoreactivities in the spinal cord under different conditions

**Figure 2.**
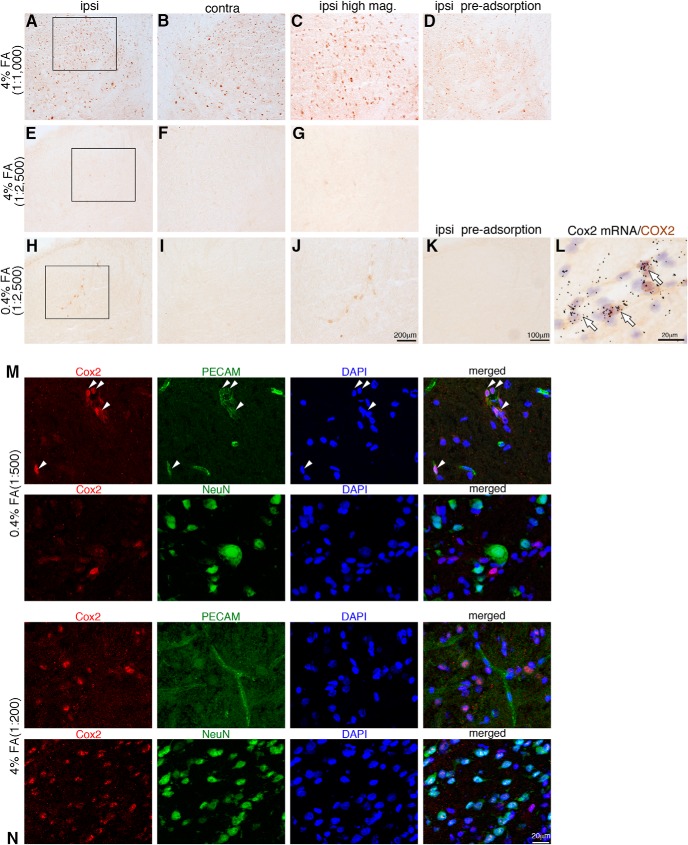
Up-regulation of COX2 in spinal endothelial cells 48 h after surgery. ***A–L***, Spinal cord sections from SNI at 48 h after surgery in the 4% FA–fixed condition with anti-COX2 antibody at 1:1000 dilution (***A–D***) and 1:2500 (***E–G***) and in the 0.4% FA–fixed condition with anti-COX2 antibody at 1:2500 dilution (***H–L***). Lower-magnification photographs show the ipsilateral (***A***, ***E***, ***H***) and contralateral (***B***, ***F***, ***I***) dorsal horn. Higher-magnification photographs from the ipsilateral dorsal horn (***C***, ***G***, ***J***). ***J*** shows COX2-ir cells in blood vessel-like cells; in contrast, the high concentration of the antibody in the 4% FA–fixed condition shows COX2-ir neurons (***A–C***). COX2 immunostaining of the ipsilateral dorsal horn using COX2 antibody preabsorbed with COX2 peptide (***D***, ***K***). ***L***, Bright-field photomicrograph showing combined ISHH for Cox2 mRNA with immunostaining of COX2 in the 0.4% FA–fixed condition 48 h after peripheral nerve injury. ***M***, ***N***, Double immunofluorescent histochemistry of COX2 (red) with PECAM1 (green) or NeuN (green), and DAPI (blue) in the 0.4% (***M***) and 4% (***N***) FA-fixed conditions 48 h after SNI. The right photographs show merged images of COX2, NeuN or PECAM1, and DAPI in the ipsilateral dorsal horn. COX2-ir signals in the 0.4% FA–fixed condition were colocalized with PECAM1 but not with NeuN (***M***); in contrast, in the 4% FA–fixed condition, COX2-ir was colocalized with NeuN but not with PECAM1 (***N***). Scale bars: ***A***, ***B***, ***D***, ***F***, ***G***, ***I***, ***J***, and ***L***: 100 µm; ***C***, ***H***, and ***K***: 200 µm; ***E***: 20 µm. Contra, contralateral; ipsi, ipsilateral.

Previous immunohistochemistry studies demonstrated that COX2 is expressed in both neurons and astrocytes (Maihöfner et al., 2000; Broom et al., 2004; Ghilardi et al., 2004; Vardeh et al., 2009), whereas ISHH studies reported that COX2 mRNA is expressed in blood vessel cells (Ichitani et al., 1997; Schuligoi et al., 2003) in the spinal cord. In addition, inflammatory mediator–dependent endothelial COX2 expression has been shown by immunohistochemistry in the damaged nervous system (Cao et al., 1996, 1998; Quan et al., 1998; Cao et al., 1999; Laflamme et al., 1999). One possible reason for this discrepancy might be the difference in methodology, especially regarding immunohistochemistry methods. Therefore, we tested the effect of different concentrations of the fixative formaldehyde (FA) on the immunohistochemistry of COX2 (see Materials and Methods; Fig. 2). First, we examined 4% FA postfixation using fresh-frozen sections. Although obvious immunoreactive (ir) signals were detected in this condition (concentration of antibody 1:1000), there was no difference in expression between the ipsilateral (Fig. 2*A*) and contralateral (Fig. 2*B*) side, and COX2-ir was observed in neuron-like cells (Fig. 2*C*) 48 h after peripheral nerve injury. A pre-adsorption test of the antibody for COX2 showed that immunoreactive cells were detected in neuron-like cells in the 4% FA–fixed condition (Fig. 2*D*). The lower concentration of antibody (1:2500) could not detect COX2-ir in the 4% FA–fixed spinal cord (Fig. 2*E–G*). We tested the perfusion method with 4% PFA fixative. The result was quite similar to that for the postfixation samples using 4% FA (data not shown). We then reduced the concentration of FA (0.4% FA) using fresh-frozen sections. In the 0.4% FA–fixed condition, COX2-ir levels increased in blood vessel–like cells ipsilateral to the nerve injury (Fig. 2*H–J*).

In contrast to the 4% FA sections, there was no immunoreactivity in the pre-adsorption section in 0.4% FA–fixed condition (Fig. 2*K*). We further performed double-labeling with ISHH and IHC for COX2 and found that COX2-ir cells completely colocalized with Cox2 mRNA signals in blood vessel cells (Fig. 2*L*). These findings suggest that COX-2-ir signals in the 4% FA–fixed condition and in the high concentration antibody condition were not specific. When double-labeling with COX2 and two marker proteins (NeuN and PECAM1) was performed, immunoreactivities in the 0.4% FA–fixed condition showed that COX2 was colocalized with PECAM1, but not with NeuN (Fig. 2*M*), which is consistent with the ISHH data. The COX2-ir signals were localized in the vicinity of the nuclear membrane (Fig. 2*M*). By contrast, COX2 was colocalized with NeuN, but not with PECAM1 in the 4% FA–fixed-condition (Fig. 2*N*). These results suggest that in these experimental conditions, Cox2 mRNA and COX2 protein expression was induced in endothelial cells in the spinal cord after peripheral nerve injury.

### PGI2 and the receptor involved in the development of neuropathic pain

**Figure 3.**
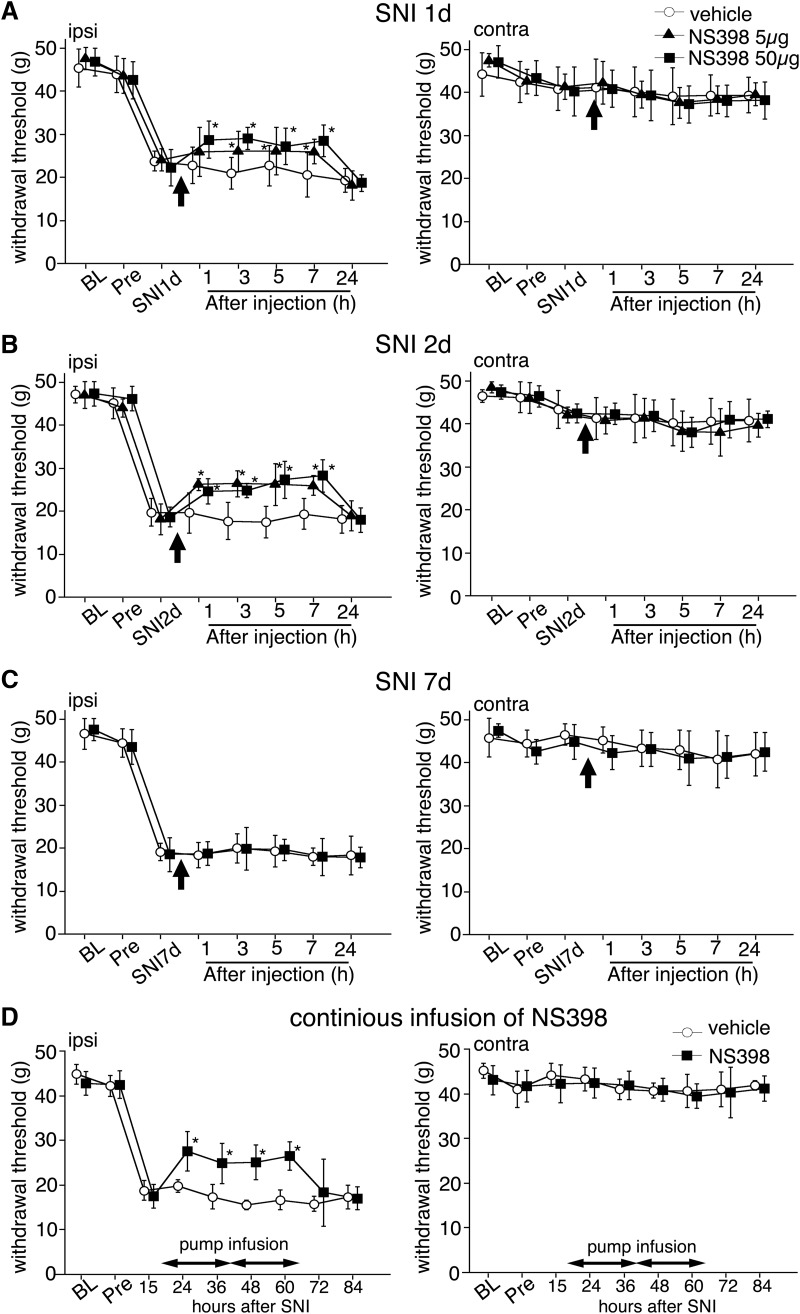
Intrathecal injection of COX2 inhibitor NS398 attenuated mechanical allodynia induced by SNI. The line graphs show effects of NS398 1 (***A***), 2 (***B***), and 7 (***C***) d after surgery. The single-shot injection of NS398 (5 µg/10 µl or 50 µg/10 µl) attenuated mechanical allodynia for 1 (***A***) and 2 (***B***) d after surgery. NS398 did not have a positive effect on mechanical allodynia 7 d after surgery (***C***). ***D***, Continuous infusion of NS398 at 5 µg/8 µl/h attenuated mechanical allodynia. There was no effect of NS398 on the mechanical pain threshold in the contralateral hindpaw (***A–D***). The tube was set at 1 d before SNI surgery. In all graphs, values are represented as mean ± SD (*n* = 5–6 in each group, *, *p* < 0.05 compared with vehicle at each time point). BL, base line (before indwelling tube surgery); pre, before SNI surgery.

**Figure 4.**
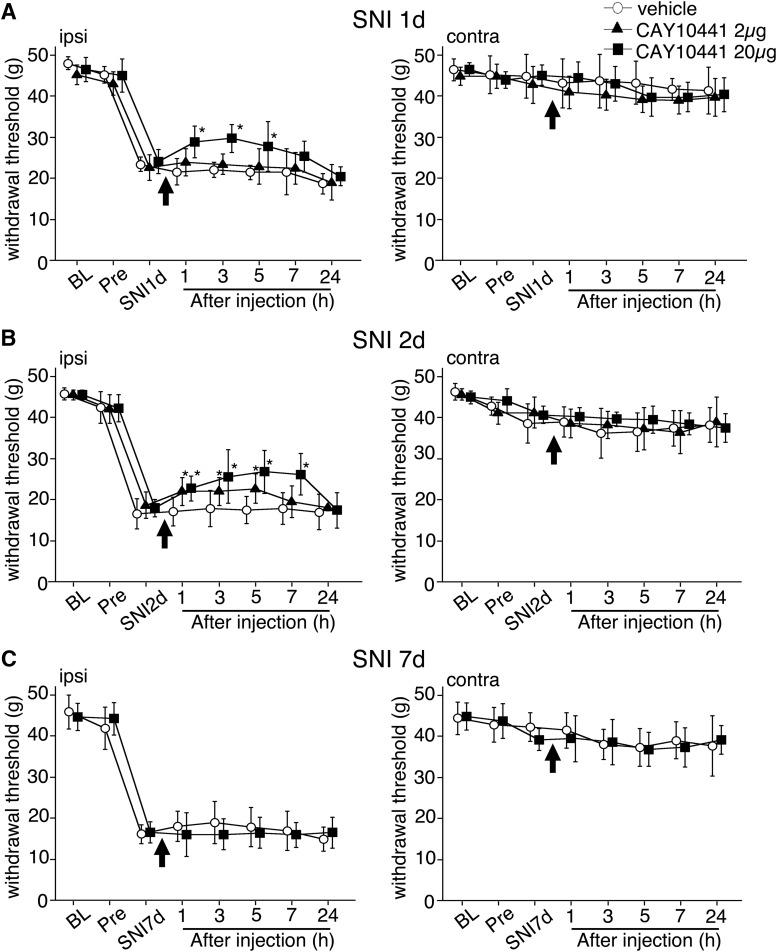
Intrathecal injection of IP receptor antagonist CAY10441 attenuated mechanical allodynia induced by SNI. The line graphs show effects of CAY10441 at 1 (***A***), 2 (***B***), and 7 (***C***) d after surgery. At 1 and 2 d after surgery, the single injection of CAY10441 (2 µg/10 µl or 20 µg/10 µl) attenuated mechanical allodynia (***A***, ***B***). CAY10441 did not rescue the mechanical pain threshold at 7 d after surgery. The tube was set at 1 d before SNI surgery. There was no effect of CAY10441 on the mechanical pain threshold in the contralateral hindpaw (***A–C***). In all graphs, values are represented as mean ± SD (*n* = 6 in each group, *, *p* < 0.05 compared with vehicle at each time point). BL, base line (before indwelling tube surgery); pre, before SNI surgery.

We examined the effect of a COX2 inhibitor and an IP receptor antagonist on mechanical hyperalgesia in an SNI model (Decosterd and Woolf, 2000). Cox2 and Pgis mRNA levels were significantly increased in the spinal cord from 24 h and peaked at 48 h after nerve injury (see Fig. 1*B*). Therefore, we investigated the time course–dependent effect of these inhibitors on pain behaviors after nerve injury. Single-shot intrathecal injection of NS398 (COX2 inhibitor; 5 µg/10 µl, 50 µg/10 µl) at day 1 and 2 significantly attenuated SNI-induced mechanical hyperalgesia until 7 h after administration (Fig. 3*A*, *B*). However, NS398 did not attenuate pain behavior at day 7 (Fig. 3*C*). CAY10441 (IP receptor antagonist; 2 µg/10 µl, 20 µg/10 µl) also attenuated pain behavior at day 1 and 2 after SNI, but not at day 7 (Fig. 4*A–C*). We furthermore examined the effect of continuous intrathecal infusion with an osmotic pump from 18 to 66 h after injury (Fig. 3*D*). Mechanical allodynia in 400 µg/h NS398 administration groups was significantly reversed 24–60 h after injury (*p* < 0.05 vs. vehicle). The treatments did not have any effect on the contralateral side. These results indicate that the inhibition of PGH2 production and IP receptor signaling attenuated the SNI-induced neuropathic pain. PGI2 may be involved in the mechanisms underlying the development of neuropathic pain after peripheral nerve injury through endothelial–neuronal interactions.

### Transient increase in TNFα mRNA via phosphorylation of p38 MAPK in the spinal cord after SNI

**Figure 5.**
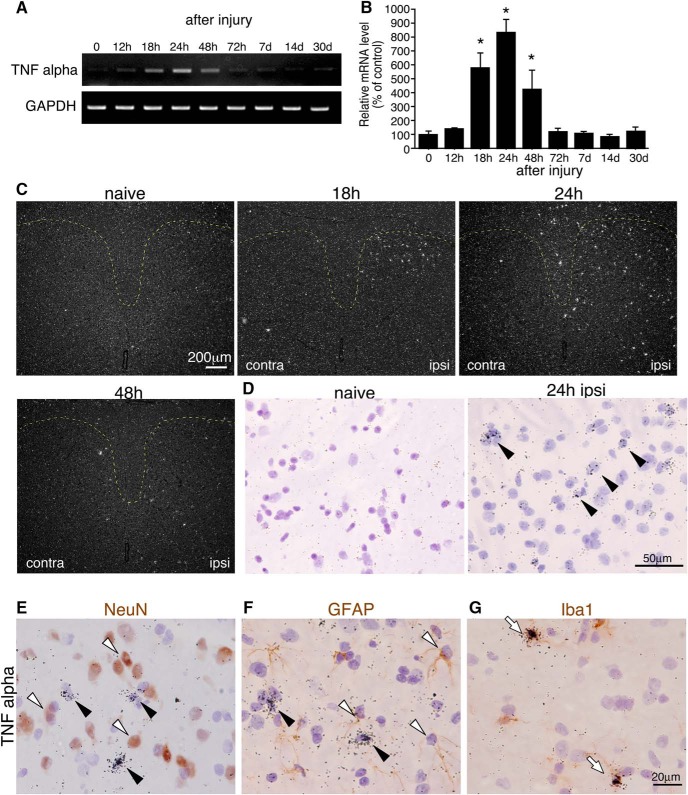
SNI up-regulates TNFα mRNA in the ipsilateral microglia. ***A***, Semiquantitative RT-PCR analysis shows TNFα mRNA expression in the L4–5 ipsilateral spinal cord taken from rats at 0 (naive), 12, 18, 24, 48, and 72 h and 7, 14, and 30 d after nerve injury. ***B***, Quantification of relative levels of TNFα mRNA. GAPDH was used as the loading control. Data were first normalized to GAPDH and then expressed as fold change compared with the naive rat spinal cord. (*n* = 5, mean ± SE, *, *p* < 0.05; **, *p* < 0.001 by Fisher’s PLSD). ***C***, ***D***, Low magnification dark-field images (***C***) and high magnification bright-field images (***D***) show the ISHH for TNFα mRNA in the dorsal horn. Arrowheads indicate positive cells. ***E–G***, Bright-field images show combined ISHH for TNFα mRNA with immunostaining of NeuN (***E***), GFAP (***F***), and Iba1 (***G***) in the ipsilateral dorsal horn 24 h after surgery. Scale bar: dark-field images (***C***): 200 µm; bright-field images (***D***): 50 µm, bright-field images (***E–G***): 20 µm. Black arrowheads indicate cells single-labeled with ISHH (aggregation of grains). Open arrows indicate cells double-labeled with ISHH and IHC.

**Figure 6.**
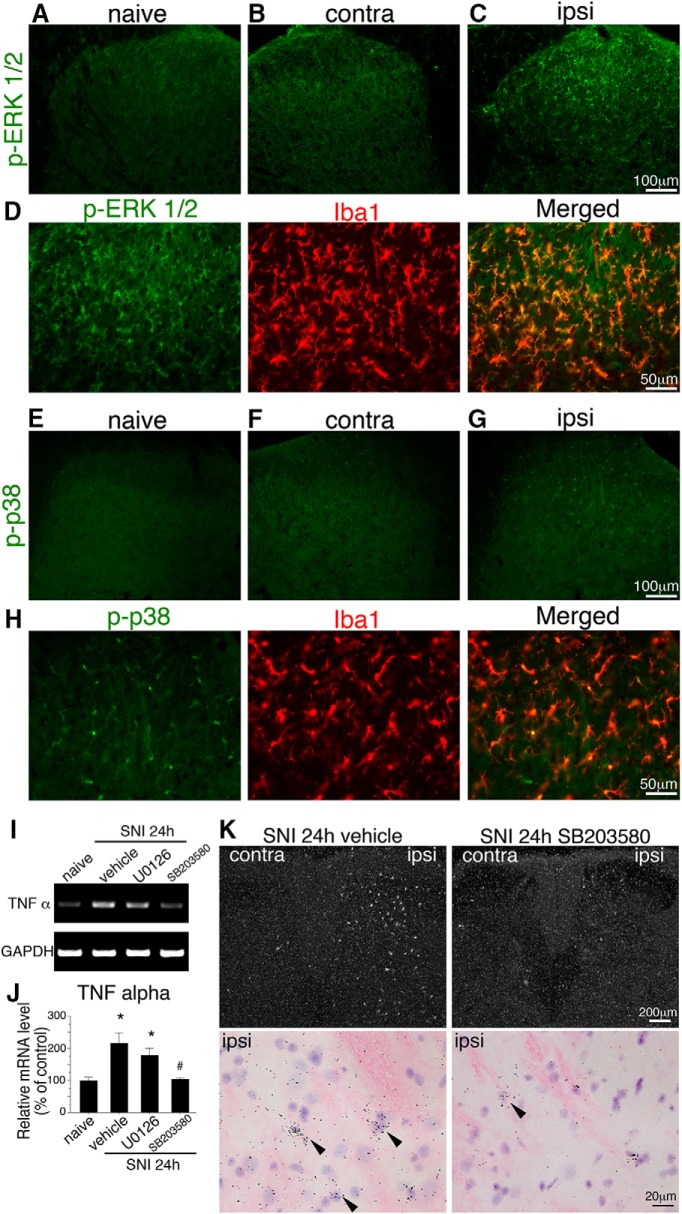
Inhibition of p38 MAPK suppressed induction of TNFα mRNA in spinal microglia after peripheral nerve injury. ***A–C***, Expression of phosphorylated ERK 1/2 in the dorsal horn: naive (***A***), 24 h after SNI contralateral (***B***), and ipsilateral (***C***), revealed by IHC analysis. Phosphorylated ERK 1/2 immunoreactivity was increased on the ipsilateral side 24 h after peripheral nerve injury, compared with the contralateral side. ***D***, Double IHC of phosphorylated ERK1/2 (green) with Iba1 (red) 24 h after SNI. Right photograph shows merged images of p-ERK1/2 and Iba1 in the ipsilateral dorsal horn. ***E***, ***F***, Expression of p-p38 in the dorsal horn: naive (***E***), 24 h after SNI contralateral (***F***), and ipsilateral (***G***). Phosphorylation of p38 immunoreactivity was increased on the ipsilateral side 24 h after peripheral nerve injury, compared with the contralateral side. ***H***, Double IHC of p-p38 (green) with Iba1 (red) 24 h after SNI. Right photograph shows merged images of p-p38 and Iba1 in the ipsilateral dorsal horn. Peripheral nerve injury induces activation of ERK1/2 and p38 MAP kinase in microglia in the spinal cord 24 h after the injury. Scale bars: ***A–C***, ***E***, ***F***: 100 µm; ***D***, ***H***: 50 µm. Contra, contralateral; ipsi, ipsilateral. ***I***, Effect of MAPK inhibitors (p38 MAPK inhibitor SB203580 and MEK inhibitor U0126) on the induction of TNFα mRNA. Gel panels show RT-PCR products from the L4–5 spinal cord taken from naive rats, 24 h after SNI, intrathecally administered with vehicle, U0126 (12 µg/d) and SB203580 (12 µg/d). ***J***, Graphs show the quantification of relative mRNA levels of TNFα normalized against GAPDH (*n* = 4–5, mean ± SEM; *, *p* < 0.05 compared with naive, #, *p* < 0.05 compared with vehicle control). ***K***, Dark-field photographs of ISHH show TNFα mRNA in the dorsal horn of SNI rats treated with vehicle and SB203580. Higher-magnification photographs from hematoxylin-eosin counterstained sections under bright-field illumination in the ipsilateral dorsal horn of vehicle and SB203580 24 h after nerve injury. Scale bars: dark-field photographs, 200 µm; bright-field photographs, 20 µm.

It has been reported that LPS, proinflammatory mediators, and growth factors induce the expression of COX2 (Tsatsanis et al., 2006). Particularly, TNFα induces COX2 expression in brain blood vessels (Cao et al., 1998; Matsumura et al., 1998; Nadeau and Rivest, 1999). To clarify the effect of TNFα on the induction of COX2 in endothelial cells, we first examined TNFα expression after SNI. We found that TNFα mRNA levels rapidly and transiently increased in the ipsilateral spinal cord after nerve injury, detectable at 18 h after surgery, and reached a peak at 24 h. TNFα mRNA levels had returned to normal levels at 72 h (Fig. 5*A*, *B*). TNFα mRNA was undetectable by ISHH in the dorsal horn of naive rats (Fig. 5*C*, *D*). TNFα-expressing cells were found in the superficial dorsal horn 18 h after injury, and the numbers of these cells increased 24 h in the ipsilateral dorsal horn (Fig. 5*C*, *D*). The majority of the TNFα mRNA–expressing cells were double-labeled with Iba1 (Fig. 5*G*) but not NeuN (Fig. 5*E*) or GFAP (Fig. 5*F*) at 24 h after nerve injury, suggesting that microglia induced TNFα. We next examined the signal transduction cascade, which is involved in the induction of TNFα, because MAPK is activated in spinal microglia after nerve injury (Jin et al., 2003; Zhuang et al., 2005). In fact, phosphorylation of the ERK1/2 and p38 MAPK was upregulated in the spinal microglia at 24 h after SNI (Fig. 6*A–H*). Therefore, we examined whether MEK inhibitor (U0126) or p38 inhibitor (SB203580) application could suppress or inhibit the upregulation of TNFα in microglia after SNI. TNFα mRNA signals were clearly decreased by the application of the p38 inhibitor SB203580, but not by U0126 (Fig. 6*I*, *J*). Next, we confirmed the effects of the p38 inhibitor on TNF-α using ISHH (Fig. 6*K*). TNFα mRNA signals were clearly decreased by the application of the p38 inhibitor SB203580. These results were consistent with those of a previous report (Mahtani et al., 2001).

### TNFR signaling regulates the transcription of COX2 and PGIS in the spinal cord

**Figure 7.**
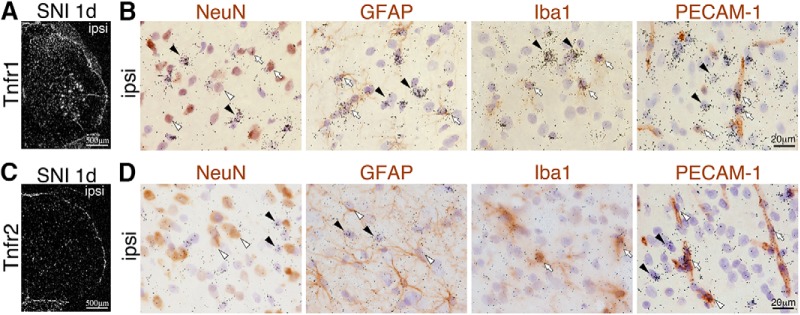
Expression pattern of TNFα receptor mRNAs in the spinal cord. ***A***, ***C***, Dark-field images of ISHH revealed the distribution of mRNA for Tnfr1 (***A***) and Tnfr2 (***C***) in the L4–5 spinal cord 1 d after injury. Scale bar: 500 µm. ***B***, ***D***, Bright-field photomicrographs showed combined ISHH for Tnfr1 (***B***) and Tnfr2 (***D***) mRNA with immunostaining of NeuN, GFAP, Iba1, and PECAM1, counterstained with hematoxylin, in the dorsal horn 1 d after injury. Open arrowheads indicate single immunostained cells (brown staining). Black arrowheads indicate cells single-labeled with ISHH (aggregation of grains). Open arrows indicate cells double-labeled with ISHH and IHC. Scale bars: 20 µm.

Two types of TNFα receptors, TNF receptor (TNFR) 1 (CD120a; p55/60) and the lower-affinity TNFR2 (CD120b; p75/80; MacEwan, 2002), have been identified, but the precise histologic expression of these receptors in the spinal cord after nerve injury has not been fully reported. To determine whether TNFα could affect endothelial cells, we investigated TNF receptor expression in the spinal cord after SNI at the day when both Cox2 and TNFα levels showed a significant increase after injury. Dark-field images in ISHH showed Tnfr mRNA–positive cells in the ipsilateral spinal cord 1 d after injury. Tnfr1 mRNA was expressed in the dorsal horn, motor neurons, ependymal cells of the central canal, and the pia mater (Fig. 7*A*), and Tnfr2 mRNA was highly expressed in the pia mater (Fig. 7*C*). To identify Tnfr mRNA–expressing cells in the spinal cord after SNI, we performed combined ISHH for Tnfr mRNA with IHC for NeuN, GFAP, Iba1, or PECAM1. Tnfr1 mRNA signal intensity was high in astrocytes, microglia, and endothelial cells (Fig. 7*B*). Tnfr1 mRNA was also detected in a small number of neurons in the dorsal horn (Fig. 7*B*). Tnfr2 was expressed in microglia and endothelial cells, but not in neurons or astrocytes 1 d after injury (Fig. 7*D*).

**Figure 8.**
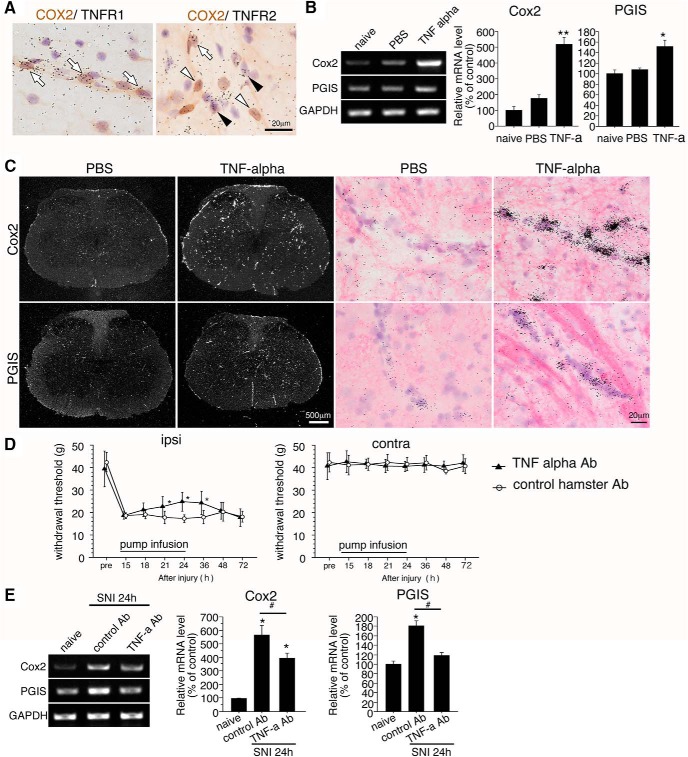
TNFα upregulates expression of COX2 and PGIS in endothelial cells. ***A***, Bright-field photomicrographs show combined ISHH for Tnfr1 and Tnfr2 mRNA with immunostaining of COX2 in the 0.4% FA–fixed condition 24 h after peripheral nerve injury. Scale bar: 20 µm. ***B***, The intrathecal TNFα injection induced Cox2 and Pgis mRNA expression. Gel panels show RT-PCR products from the L4–5 spinal cord taken from naive rats, 3 h after intrathecal injection of PBS and recombinant TNFα. The graphs showed quantification of relative mRNA levels of Cox2 and Pgis. The data were first normalized to GAPDH and then expressed as fold change compared with the naive rat spinal cord (*n* = 5, mean ± SE, *, *p* < 0.05; **, *p* < 0.001, Fisher’s PLSD). ***C***, Dark-field photographs of ISHH show Cox2 and Pgis mRNA in the spinal cord 3 h after injection of PBS or recombinant TNFα. Right higher-magnification photographs are from hematoxylin and eosin–counterstained sections under bright-field illumination in the ipsilateral dorsal horn. Scale bars: dark-field photographs, 500 µm; bright-field photographs, 20 µm. ***D***, Effects of the neutralizing TNFα antibody on neuropathic pain behaviors. Intrathecal administration of the neutralizing TNFα antibody suppressed the mechanical allodynia induced by SNI. In these graphs, values are represented as mean ± SD (*n* = 6–7 in each group, *, *p* < 0.05 compared with vehicle at each time point). Pre, before SNI surgery. ***E***, Effect of neutralizing TNFα antibody on the induction of Cox2 and Pgis mRNA. Gel panels show RT-PCR products from the L4–5 spinal cord taken from naive rats, 24 h after SNI intrathecally administered with control antibody and neutralizing TNFα antibody (0.25 µg/8 µl/h). Graphs show the quantification of relative mRNA levels of Cox2 and Pgis, normalized against GAPDH (*n* = 5, mean ± SEM; *, *p* < 0.05 compared with naive, #, *p* < 0.05 compared with control antibody).

To confirm whether TNFα induces the expression of COX2 and PGIS, we used combined ISHH for the mRNA of both TNF receptors with IHC for COX2. After SNI, double-labeled ISHH demonstrated that most COX2-ir cells contained Tnfr1 mRNA, and only part of the COX2-ir cells contained Tnfr2 mRNA (Fig. 8*A*). To determine whether TNFα mediates the up-regulation of Cox2 and Pgis mRNA in naive spinal cord, we performed intrathecal injection of recombinant TNFα and measured mRNA levels using RT-PCR. Compared with PBS injection, TNFα significantly increased the production of Cox2 and Pgis mRNA 3 h after injection (Fig. 8*B*). Then we examined the distribution of increased mRNA by ISHH and found that dark-field images showed increased signals of Cox2 and Pgis mRNA in the spinal cord and the pia mater after TNFα application. Bright-field images showed that both mRNAs were expressed along the blood vessels (Fig. 8*C*). These results indicate that TNFα is a key mediator that can lead to the induction of COX2 and PGIS expression in endothelial cells after nerve injury. Considering that TNFα levels increased in spinal microglia, and considering the effects of TNFα on TNFR1 and R2 in endothelial cells, the subsequent signaling of TNF receptors may increase COX2 and PGIS expression.

To examine whether TNFα contributes to mechanical allodynia after nerve injury and affects COX2/PGIS expression, we injected neutralizing TNFα antibody into SNI model rats. TNFα antibody was administered intrathecally via osmotic pumps for 24 h through a cannula implanted at the lumbar enlargement. Intrathecal injection of TNFα antibody suppressed SNI-induced mechanical allodynia 21–36 h after injury (Fig. 8*D*). Furthermore, an intrathecal injection of neutralizing TNFα antibody significantly suppressed Cox2 and Pgis mRNA levels, which were increased by nerve injury in the spinal cord (Fig. 8*E*). However, Cox2 mRNA levels were still significantly increased by neutralizing TNFα antibody treatment, compared with naive rats. Taken together, our findings suggest that COX2-dependent PGI2 production in endothelial cells is involved in neuropathic pain, and that these molecules are upregulated by TNFα released from microglia after nerve injury.

## Discussion

**Figure 9.**
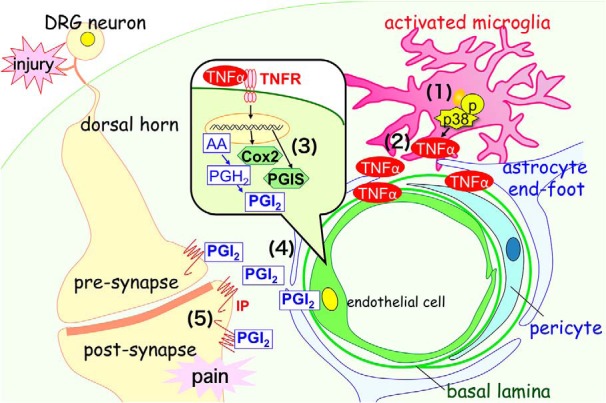
Schematic of microglia/endothelial cell/neuron crosstalk supporting developmental neuropathic pain in the spinal dorsal horn. ***1***, Nerve injury induces TNFα in microglia via p-38 MAPK from 18 to 24 h after injury. ***2***, TNFα is released from microglia. ***3***, TNFα induces COX2 and PGIS expression in endothelial cells through TNFR1/TNFR2 signaling from 24 to 48 h after nerve injury. ***4***, PGI2 is released from endothelial cells. ***5***, PGI2 binds to IP receptor in spinal neurons and is involved in neuropathic pain.

In the present study, we provide evidence for a critical role of endothelial PGI2 via COX2 in neuropathic pain mechanisms. TNFα is synthesized through p38 activation in microglia and induces COX2 and PGIS expression (Fig. 9) in endothelial cells in the spinal cord. The IP receptor in spinal neurons may contribute to increased pain behavior after the binding of PGI2 from endothelial cells. These findings suggest a novel three-way interaction among microglia, endothelial cells, and neurons in the mechanisms underlying neuropathic pain after peripheral nerve injury.

It is well established that COX1 is a constitutive enzyme, whereas COX2 is an inducible enzyme that mediates inflammation and pain (Seibert et al., 1994; Simon, 1999); peripheral inflammation upregulates the expression of COX2 in the spinal cord (Ichitani et al., 1997; Tegeder et al., 2001). However, there is a discrepancy regarding the localization of COX2 in the spinal cord after peripheral inflammation; COX2 was reported to be expressed in neurons by IHC (Samad et al., 2001) or blood vessels by ISH (Ichitani et al., 1997). Samad et al. (2001) reported that COX2 was upregulated in dorsal horn neurons after peripheral inflammation, and that a COX2 inhibitor could reduce pain behavior.

It has been reported that after peripheral nerve injury, COX2 was not induced in the spinal cord that and a COX2 inhibitor and nonsteroidal anti-inflammatory drugs had no prominent effects on established neuropathic pain (Broom et al., 2004; Vo et al., 2009). In contrast, the interesting finding of our previous study was that the expression of COX2 is upregulated in blood vessels (Kanda et al., 2013); however, the detailed expression pattern and whether COX2 is involved in the pathogenesis of neuropathic pain were unclear at the time. In this study, we demonstrated by semiquantitative RT-PCR, that Cox2 and Pgis mRNA were induced during a short period in the early phase of neuropathic pain. We showed induction of Cox2 in endothelial cells after nerve injury using ISHH and found two staining patterns of COX2 with different concentrations of fixing solutions using IHC: immunoreactivity in blood vessels (0.4% FA–fixed condition) and in neurons (4% FA–fixed condition). It is well known that fixation conditions could affect staining in IHC, and previous studies have reported that sub- or supra-optimal fixations increase nonspecific signals or lead to the loss of immunoreactivity (Volk et al., 2005; Lorenzo et al., 2014). We believe that in the case of the antibody we used in the present study, a low concentration of FA might be more specific, because it was consistent with the data in ISHH and because the signals of Cox2 mRNA with ISHH were completely colocalized with COX2 immunoreactivities.

Pgis mRNA levels increased in endothelial cells after nerve injury and completely colocalized with induced COX2-ir cells. It is well known that PGIS is localized in endothelial cells and vascular smooth muscle cells (Majed and Khalil, 2012). In addition, COX2 but not COX1 inhibitor suppressed PGI2 production in cultured human endothelial cells (Caughey et al., 2001) and whole blood vessels *ex vivo* (McAdam et al., 1999). These results corroborate our results, indicating that PGI2 is synthesized in spinal endothelial cells via COX2 after nerve injury. In terms of pain behavior, mechanical allodynia after intrathecal injection of COX2 was attenuated by the selective antagonist NS-398. However, once the neuropathic pain was established, NS-398 showed no effect on pain behavior. The critical period for application of NS-398 was thus limited to the early phase of neuropathic pain development. We therefore suggest that COX2 and PGIS were upregulated in endothelial cells in the spinal cord after peripheral nerve injury, and that both might play important roles in the early phase of neuropathic pain.

Previous reports showed that PGI2 plays a role in pain and inflammation via the IP receptor under pathologic conditions (Murata et al., 1997; Schuh et al., 2013). The IP receptor is known to couple with Gs, Gq, or Gi proteins (Lawler et al., 2001). The pronociceptive role of the IP receptor in peripheral mechanisms has been well established, and IP receptor mRNA is expressed in small- and medium-sized DRG neurons (Oida et al., 1995), which was confirmed by our experiment in ISHH (data not shown). PGI2-IP signaling sensitized transient receptor potential vanilloid type 1 (TRPV1) in a protein kinase (PKC and PKA)-dependent manner in DRG neurons (Namba et al., 1994; Moriyama et al., 2005). In the spinal cord, the IP receptor is expressed in dorsal horn neurons, and it colocalized with glycine receptor alpha 3 (Schuh et al., 2013). An autoradiographic study reported a high density of binding sites for the PGI2 analog [^3^H]iloprost in the dorsal horn, and that dorsal rhizotomy decreased binding site density in the dorsal horn (Muramatsu et al., 2012). In a model of local experimental autoimmune encephalomyelitis, PGI2 derived from spinal endothelial cells was found to be involved in axonal remodeling (Muramatsu et al., 2012). We also confirmed signals for IP receptor mRNA in spinal neurons, and found no changes after nerve injury. An intrathecal injection of IP receptor–selective agonist cicaprost has been shown to induce mechanical allodynia (Doi et al., 2002). Considering all these findings, we suggest that COX2-dependent PGI2 from endothelial cells affects neuronal excitability via the IP receptor expressed in dorsal horn neurons and primary afferents, and that endothelial cells might play a role in the modulation of pain signaling in the central nervous system. We have to note that the IP receptor antagonist CAY10441and the COX2 inhibitor NS398 had significant inhibitory effects only during the early phase of neuropathic pain, and that the antagonism of COX2 and PGI2 transiently and modestly altered the withdrawal thresholds of mechanical hypersensitivity.

TNFα is a proinflammatory cytokine, and a number of reports have indicated its involvement in pain sensitization in the central nervous system (Leung and Cahill, 2010). An intrathecal injection of TNFα leads to mechanical allodynia (Park et al., 2011), and TNFα has many regulatory functions in pain pathways: it enhances the tetrodotoxin-resistant Na^+^ current in DRG neurons (Jin and Gereau, 2006; Czeschik et al., 2008), regulates spinal cord synaptic plasticity via TRPV1-activation and glutamate release (Park et al., 2011), modulates central sensitization via NMDA-induced currents (Zhang et al., 2011), and increases MCP-1 levels in astrocytes via the activation of JNK (Gao et al., 2009, 2010). In this study, we found that *Tnfr1* mRNA is expressed constitutively in dorsal horn neurons, astrocytes, microglia, and endothelial cells. TNFα signaling may be able to modulate pain sensitivity by changing dorsal horn excitability through activation of TNFα receptors in several types of cells in the dorsal horn. TNFα can affect the BBB in response to brain damage. Indeed, microglia-derived TNF leads to an increase in BBB permeability in the inflammation state (Abbott et al., 2006). Changes in BSCB permeability have been demonstrated to be critical for the initiation of neuropathic pain behaviors (Beggs et al., 2010; Echeverry et al., 2011). In addition, TNFR signaling regulates gene expression in endothelial cells after peripheral inflammation. In previous reports, TNFα has been shown to stimulate the expression of COX2 (Cao et al., 1998; Trickler et al., 2005), intercellular adhesion molecule 1, and vascular cell adhesion molecule 1 through the activation of NF kappaB (Zhang et al., 2013). In this study, we found the expression of TNFα was transiently upregulated in microglia via p-38 activation just before the induction of Cox2 and Pgis mRNA in endothelial cells. We also found that Tnfr1 and Tnfr2 were expressed in endothelial cells, and that COX2 immunoreactive cells mainly colocalized with the signals of Tnfr1 with ISHH. Interestingly, TNFα mRNA expression had returned to normal levels 72 h after nerve injury; COX2 and PGIS expression returned to normal levels as well. Some reports have shown that TNFR1 signaling regulates the transcription of inflammatory genes, and that TNFR1 is involved in neuropathic pain (Sommer et al., 1998; Amrani et al., 2001). We found that intrathecal injection of TNFα rapidly induced the expression of Cox2 and Pgis mRNA in blood vessels, whereas the application of TNFα neutralizing antibody attenuated mechanical allodynia and the induction of Cox2 and Pgis mRNA after nerve injury. Therefore, we suggest that TNFα from microglia is a key molecule that initiates the production of PGI2 in endothelial cells after peripheral nerve injury. The initiation of the production of PGI2 may be a previously unknown interaction between microglia and endothelial cells in the spinal cord after peripheral nerve injury.

## Synthesis

Reviewing Editor: Karen Davis, Krembil Research Institute and University of Toronto Decisions are customarily a result of the Reviewing Editor and the peer reviewers coming together and discussing their recommendations until a consensus is reached. When revisions are invited, a fact-based synthesis statement explaining their decision and outlining what is needed to prepare a revision will be listed below. The following reviewer(s) agreed to reveal their identity: Andrew J. Todd. Note: If this manuscript was transferred from JNeurosci and a decision was made to accept the manuscript without peer review, a brief statement to this effect will instead be what is listed below.

This article was originally submitted to the Journal of Neuroscience, and in this resubmission the authors have addressed most of the points raised by the original reviewers. The study has been performed with considerable care, and the results are very convincing.

I feel that a few minor changes are still needed:

line 153: presumably this should be “0.4% or 4% . . .”, since sections were fixed with only one of these concentrations.

line 164: it would be better to define the amount of blocking peptide in terms of molarity, rather than weight.

lines 175, 176, 213 and 215: “antiserum” has not been corrected to “antibody”.

lines 196, 198: there is no need to repeat the word antibody in these 2 lines - the first of each pair should be omitted.

line 208: presumably this part of the study was done with fresh frozen (rather than perfusion-fixed) tissue. This should be clarified.

lines 415-420: I accept that quantification of the expression of Tnfr1 and 2 in different cell types may be difficult, but it is not really acceptable to make claims concerning cell type-specific changes without some evidence. I feel that the authors should explain why they did not do this, and tone down the statements about these changes.

Although the quality of the English means that the article can be understood, I feel that it would benefit from being proof read by a native speaker.
